# Body movement as a biomarker for use in chronic pain rehabilitation: An embedded analysis of an RCT of a virtual reality solution for adults with chronic pain

**DOI:** 10.3389/fpain.2022.1085791

**Published:** 2022-12-20

**Authors:** Sammeli Liikkanen, Mika Mäkinen, Teppo Huttunen, Toni Sarapohja, Carina Stenfors, Christopher Eccleston

**Affiliations:** ^1^ R&D, Orion Corporation Orion Pharma, Turku, Finland; ^2^ Estimates PLC, Turku, Finland; ^3^ Centre for Pain Research, The University of Bath, Bath, United Kingdom; ^4^ Department of Clinical and Health Psychology, University of Ghent, Ghent, Belgium; ^5^ Department of Psychology, University of Helsinki, Helsinki, Finland

**Keywords:** chronic low back pain (CLBP), digital therapeutics, virtual reality, fear of movement/(re)injury, ROHKEA, VIRPI

## Abstract

**Introduction:**

Chronic low back pain (CLBP) is a major public health problem. Reliably measuring the effects of chronic pain on movement and activity, and any changes due to treatment, is a healthcare challenge. A recently published paper demonstrated that a novel digital therapeutic (DTxP) was efficacious in reducing fear of movement and increasing the quality of life of adult patients with moderate to severe CLBP. In this paper, we report a study of how data from wearable devices collected in this study could be used as a digital measure for use in studies of chronic low back pain.

**Methods:**

Movement, electrodermal recording, general activity and clinical assessment data were collected in a clinical trial of a novel digital therapeutic intervention (DTxP) by using the sensors in commercial Garmin Vivosmart 4, Empatica Embrace2 and Oculus Quest wearables. Wearable data were collected during and between the study interventions (frequent treatment sessions of DTxP). Data were analyzed using exploratory statistical analysis.

**Results:**

A pattern of increased longitudinal velocity in the movement data collected with right-hand, left-hand, and head sensors was observed in the study population. Correlations were observed with the changes in clinical scales (Tampa Scale of Kinesiophobia, EQ5D Overall health VAS, and EQ5D QoL score). The strongest correlation was observed with the increased velocity of head and right-hand sensors (Spearman correlation with increasing head sensor velocity and Tampa Scale of Kinesiophobia −0.45, Overall health VAS +0.67 and EQ5D QoL score −0.66). The sample size limited interpretation of electrodermal and general activity data.

**Discussion/Conclusion:**

We found a novel digital signal for use in monitoring the efficacy of a digital therapeutics (DTxP) in adults with CLBP. We discuss the potential use of such movement based digital markers as surrogate or additional endpoints in studies of chronic musculoskeletal pain.

**Clinical Trial Registration:**

https://clinicaltrials.gov/ct2/show/NCT04225884?cond=NCT04225884&draw=2&rank=1, identifier: NCT04225884.

## 1. Introduction

Data from external sensors provide opportunities for the monitoring of disease and response to treatments within both clinical trials and observatory studies (1–3).

The measurement of pain and its deleterious effects on multiple domains of life is a clinical and research challenge (4). Pain is a private mental event unobservable except by the sufferer (5). There is no objective measure of a subjective experience, but there can be objectively defined correlates of that experience, measured physiologically (6–8), through observation (9), or by the capture of alterations in gross or fine movement of body or limbs (10, 11).

Advances in technology have increased the clinical use cases as sensors become more usable and able to accurately measure various signals (12). From vital signs (over time) to movement (fine and gross body and limb position in space), geolocation, steps, and physiological activity such as heart rate, heart rate variability, skin conductance and sleep (6, 7, 13, 14). In neurology, cognitive function has also been explored, often with gamification, for its potential to provide clinically valid digital signals (15–17).

Digital biomarkers in chronic pain can usefully focus on the cognitive, emotional, relational, or physical domains. Chronic low back pain is often characterized by the avoidance of movements believed to put one at risk of further pain or re-injury (18). Capturing the extent of movement restriction, and its potential restoration in treatment, is a valid mechanistic target for a clinical endpoint in both trials and clinical practice.

In this study, we made use of a pilot clinical trial of a novel digital therapeutic intervention (DTxP) tested on adults with chronic low back pain and high levels of disability and fear of movement and re-injury, known as the VIRPI trial. The study was conducted fully remotely in 2020 and 2021. The Virtual Reality (VR) intervention was effective in reducing fear of movement, and in improving subjectively reported quality of life when compared to a sham VR comparator and treatment as usual (19). Important here is that adults experienced about 30 daily sessions of treatment. This repetitive exposure to movement in a trial environment gave a unique opportunity to explore movement data collected by VR devices and two wearables. Our first goal was exploratory: to establish routines and protocols for managing multiple data sources from different devices over a long rehabilitation intervention with repetitive movements. More specifically, our second goal was to identify movement behaviors that correlate with clinical data and consider their use as digital endpoints. We had no a-priori hypotheses as to which specific movement(s) might emerge as important, and so adopted a data-driven approach.

## 2. Materials and methods

### 2.1. Procedure

The VIRPI study was registered on ClinicalTrials.gov (NCT04225884). The study design was a double blind three-arm prospective, double-blind, randomized controlled trial comparing a digital therapeutics software solution for chronic pain (DTxP), a Sham placebo comparator, both against standard care (19). Adults with chronic low back pain were recruited from the community between January 2020 to October 2020, screened and then randomly allocated to one of the study arms. Participants and study personnel were blind to allocation.

The DTxP was a fully immersive VR experience with 24 modules (= individual days of intervention) over 6–8 weeks. It took place in a lakeshore environment, and had e.g., behavior change content provided by a virtual disembodied mentor, and physiotherapeutically designed gamified tasks (19). The Sham placebo comparator was the same VR environment as in the DTxP arm however it contained no behavior change content (19). These participants were advised to relax while in the same virtual environment as with DTxP. The sham intervention controls for non-specific influences such as expectations of treatment and aspects of delivery such as duration of exposure, environment, immersion, etc. (20). We assessed that the immersion itself will have an intrinsic effect on the outcome. As the participants in both VR arms were using the same device and the immersive environment, the actual therapeutic content being the only difference, and neither the study participants nor the personnel knew which arm the participants belonged to, we believe the term Sham placebo is justified here.

DTxP (*n* = 12) and Sham placebo (*n* = 17) participants received Oculus Quest VR head mounted device (HMD) and two handheld controllers (HHC). 10 participants were allocated to the standard care control. All (*n* = 39) received Empatica Embrace2 and Garmin Vivosmart4 wearables, and a mobile phone for data collection. Participants received written user instructions and were trained by study personnel. All were unable to see any results from the wearables (configured not to show data). Those who had their own wearable health or activity monitoring devices were allowed to continue their use.

In the data analysis we used the data from different arms as follows:

- Movement data: DTxP (*n* = 12)
- EDA: DTxP and Sham Placebo (*n* = 29)
- Activity data: DTxP, Sham Placebo and Standard Care (*n* = 39).

### 2.2. Participants

39 participants were randomized to the treatment arms. They were 34 women and 5 men, all adults with an average age of 54.7 years, with 30 out of 39 having more than 5 years of low back pain, and a mean pain intensity (on a scale of 0–5) at the start of trial of 2.8. They reported high levels of disability on the Oswestry Disability Index (ODI), with a mean of 36.1 (range 18–60), and a strong belief that movement would lead to further pain and reinjury as measured by the Tampa Scale of Kinesiophobia (TSK), with a mean of 41.8 (range 29–55).

### 2.3. Devices and data

#### 2.3.1. Movement data

The accelerometer data from the Oculus HMD and HHC devices in DTxP arm were collected when using the DTxP (sampling rate of 30 Hz). We used data from DTxP arm only, as that was the only arm where the movements related to the therapeutic content were done. The Sham placebo arm was assessed to contain a limited amount of relevant movement data, as the participants were instructed to relax in the VR environment. The software to pull the movement data from HMD and hand controllers was developed for this purpose using Unity development framework version 2019.4.18f1. The movement data were sent to backend server *via* RESTful API calls. Data from the backend server were exported in JSON format for analysis, which were transformed into CSV files by using SAS software, for analysis using R software. The use of DTxP included various activities and tasks. All intervention days began by entering a specifically designed VR environment, which after two additional virtual spaces were used to facilitate psychological and physical exercises. Some days contained psychological content, while the others contained gamified physical, psychological, and cognitive exercises, specifically designed to promote fine and gross motor movements, typically avoided by low back pain patients.

Eleven participants experienced more than 30 days in treatment, and one less than 10 days. Data from the participant with missing study days was kept in the dataset to analyze robustness and bias, the correlation results are presented both with and without this participant. There were over one million lines of data per participant, with x, y, z coordinates of three accelerometers each, resulting in over 10 million individual data-points for each participant. The data included some non-natural movement patterns, such as dropping a controller. Furthermore, some data segments did not end as expected, possibly due to suddenly turning off the VR system, or system malfunction.

Data were segmented according to VR software's metadata tags (labels explaining what sequence of program was run). However, the exact task (e.g., grabbing a virtual object) was not known and tags were just to indicate the beginning and the end of a sequence. As the movements were recorded regardless of whether the participant was given a task or not, these segments were classified as action and no-action. Furthermore, no-action segments were classified as “start of day”, “between” and “end of day”, depending on the relation to the action segments.

#### 2.3.2. Electrodermal data

Empatica Embrace 2 electrodermal activity (EDA) data were collected throughout the study (sampling rate 4 Hz). Raw and aggregated data were downloaded from the Empatica server after study finalization. For the analysis of EDA data, both DTxP and Sham placebo arms were used. Data were collected during the treatment, as with the movement data. In DTxP arm, the participants performed movement related tasks, while no tasks were instructed in the Sham arm. For the analysis of DTxP arm, the segmentation of the EDA data was used to distinguish between action and no-action segments. DTxP data before first action, the start of the day segments, were removed from the EDA analysis to compare active phase of DTxP to no-action Sham data, as the Sham arm data were assumed to consist of fully no-action segments.

#### 2.3.3. Activity data

All participants wore the Garmin device throughout the study to collect daily activity data. Aggregated data for heart rate, steps and sleep duration were downloaded from the Garmin server after the finalization of the study.

#### 2.3.4. Clinical endpoints

##### 2.3.4.1. Fear of movement and re-injury

Study participants completed the Tampa Scale for Kinesiophobia, which includes 17 items assessing beliefs about pain-related movement and possible further pain and reinjury using a 4-point Likert scale from strongly agree to strongly disagree. Higher scores indicate higher fear of movement and re-injury.

##### 2.3.4.2. EuroQoL VAS for Overall Health

Participants reported their overall health condition using a visual analogue scale from 0 to 100 anchored with 0 as “the worst health you can imagine” and 100 as “the best health you can imagine”.

##### 2.3.4.3. Quality of life (QoL)

Participants completed the European Quality of Life 5- dimension, 5-level scale (EuroQoL-5D-5L). Five dimensions are assessed including mobility, self-care, usual activities, pain/discomfort, and anxiety and depression. Each item is scored from 1 to 5 (1 = no problems; 5 = unable to/extreme problems).

### 2.4. Statistical methods

#### 2.4.1. Movement data

Previous research in patients with CLBP have shown that motor and problem-solving skill exercises work well in improving cognitive impairment (21–23). Furthermore, increased activity has shown to correlate negatively with the chronic pain, and the movement velocity has shown to correlate with functional recovery in chronic pain (24, 25). Thus, we used an assumption that the time spent on a given activity correlates with the condition of a participant, i.e., a healthy participant would finish the activities faster than a participant with CLBP. As the precise nature of the activity within VIRPI segments was unavailable, the segment average velocity was used to assess how quickly participants completed their tasks. Faster average velocity indicates finishing the activity faster.

Movements were collected from VR controllers that all recorded x-, y- and z-coordinates. Time interval between recordings is approximately 0.03 s, with some variation over time. These timepoints were then combined to segments of movement based on metadata. To analyze the movement data, the coordinates of the controllers were transformed into the velocity in three-dimensional space. Velocity in every timepoint was calculated as change from previous timepoint:

$$\frac{\sqrt{\left(\Delta x^{2}+\Delta y^{2}+\Delta z^{2}\right)}}{\Delta \mathrm{time}}$$

Velocity calculated in every timepoint was then aggregated to the average velocity for every movement segment.

To validate the segmentation, velocity distributions of action segments and no-action segments were compared visually. For further analysis, only data labeled as action were used as our focus was the effects of movement. To estimate participants' progress over time, linear regression lines were fitted to segment averages over study days. Slope of regression was assessed, and positive slope considered as increase of velocity over time and negative as decrease of velocity over time. Linear fit was not an optimal fit for the data, but it was kept for simplicity and for a need to get a single measure to describe the direction of change over the time. Some study days span over multiple calendar days due to technical or other problems during activities and there were planned pauses in the activity schedule. This means that the diurnal aspect of regression is not naturally linear and therefore results of regression should be regarded only as proxy for natural within day progression of the participants.

To assess the association of movement data and clinical endpoints, correlation between the change in the velocity of movement controllers (Head, Left, Right) and change in clinical measurements (TSK, Overall health VAS, EQ-5D-5L QoL score) was calculated. For controllers, the slope of the regression line was used as the measure of change. The slope represents the estimate of daily change, so for correlation calculations the slope was multiplied by the count of participants' VR study days, thus representing the estimate of change in movement over time in study. For Clinical measurements the change was measured as the change from baseline at End of Treatment after 30 study days. For robustness, the correlations were assessed with the full data and after removing the participant with 2/3 of missing study days. Correlations were calculated as parametric and non-parametric, as with limited number of samples single measurements can have large effect on the parametric results.

#### 2.4.2. Electrodermal activity (EDA) data

There are multiple ways of analyzing EDA signals (26). It is known that EDA tends to rise when activity level rises, thus we wanted to examine this aspect. The assumption was there would be higher level of EDA from participants in the DTxP arm compared to the Sham arm.

**Figure 1:**
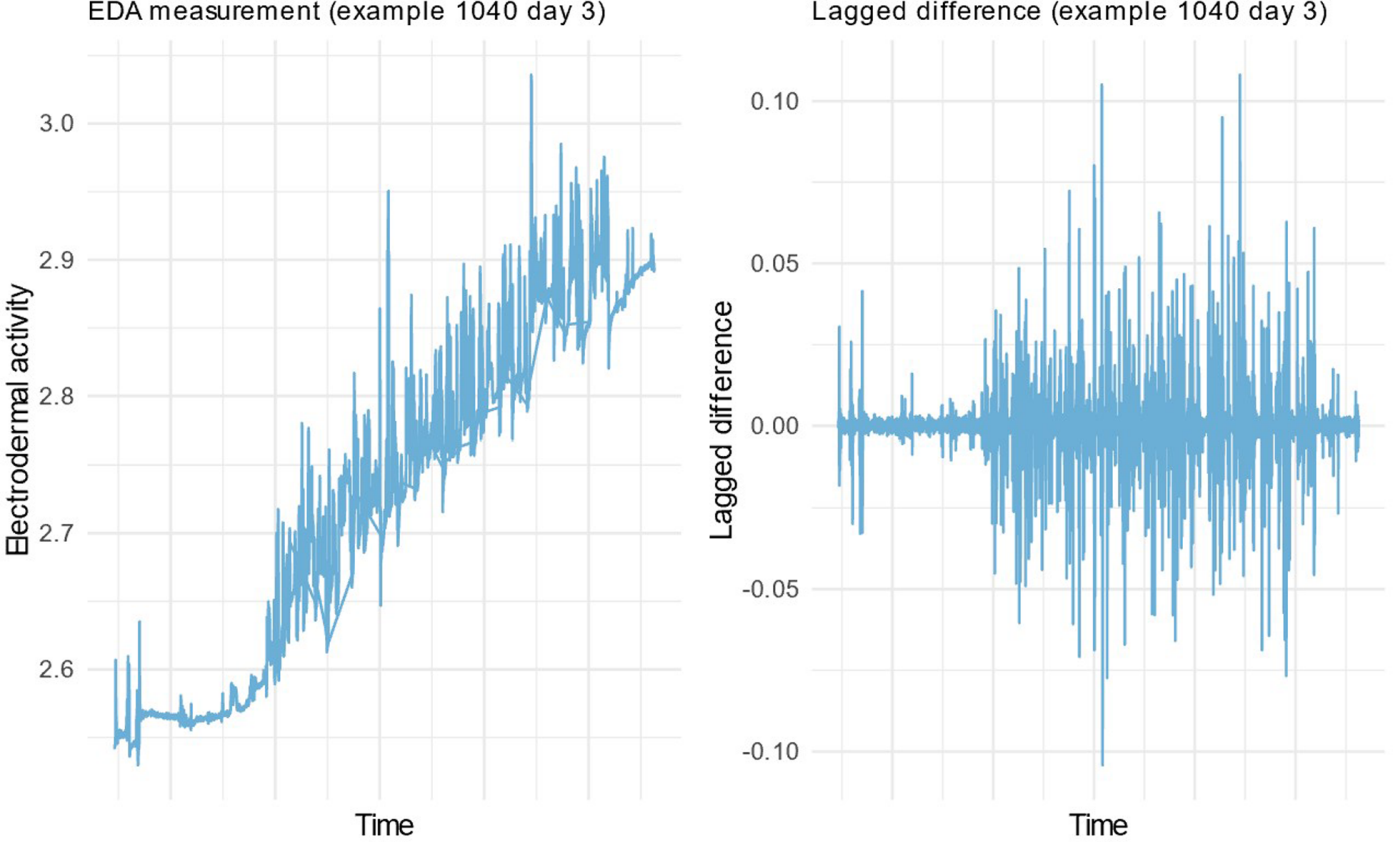
Example EDA signal with Tonic and Phasic components (left) and with the Phasic component only (right).

EDA signal is typically separated to “tonic” and “phasic” components (“tonic” is the slow and “phasic” is the faster signal variance). For this analysis we chose to concentrate on the “phasic” variance. To remove the “tonic” component, we decided to analyze difference in two consecutive timepoints (t2 – t1 = Δ) of the signal. Using the difference makes the signal stationary, as can be seen in Figure 1. To measure the overall activity in “phasic” changes, we counted the peaks of *Δ* in a segment and formed a peaks/minute measure. To distinct high peaks per minute, we formed bands using median absolute deviation (MAD):

$$\mathrm{MAD}=\mathrm{Median}\left(\left|x_{i}-\mathrm{Median}\left(x_{1..n}\right)\right|\right)$$

MAD is a robust measure of variability and tends to work better in outlier detection than standard deviation. Here we wanted to detect peaks that are equivalent to outliers in e.g., quality control situation. Peaks were defined as Δ higher than median by 2-fold MAD. Peaks were normalized by counting ratio for average peaks per minute:

$$\frac{\mathrm{Peaks}}{\mathrm{Minute}}=\frac{\mathrm{count}\left(\Delta x>2\cdot \mathrm{MAD}\left(x\right)\right)}{\mathrm{Segment}\;\mathrm{Length}\;\mathrm{in}\;\mathrm{Minutes}}$$

**Figure 2:**
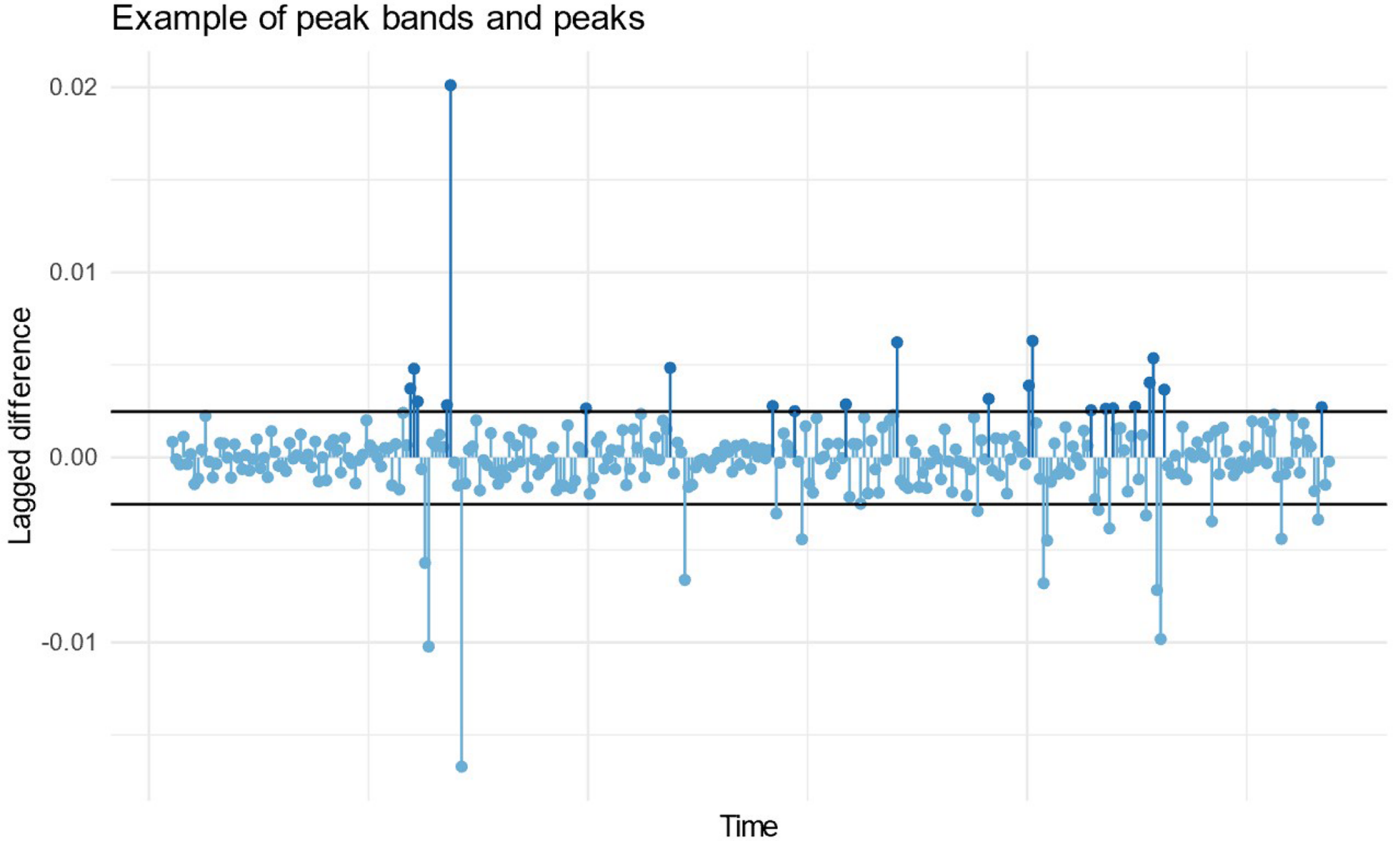
Example of a peak band and the peaks (dark blue markers) in a random sample of EDA data over couple of minutes.

A visualization of a peak band and the peaks from a random EDA data sample is shown in Figure 2. Peak count per minute distributions were compared between different groups using a visual inspection.

#### 2.4.3. Activity data

In the analysis of general activity, our approach was first to visualize the distributions between trial arms over time. Next, a regression coefficient model for the effects of group and day with each participant having an own regression line terms was assessed.

## 3. Results

### 3.1. Movement data

**Figure 3:**
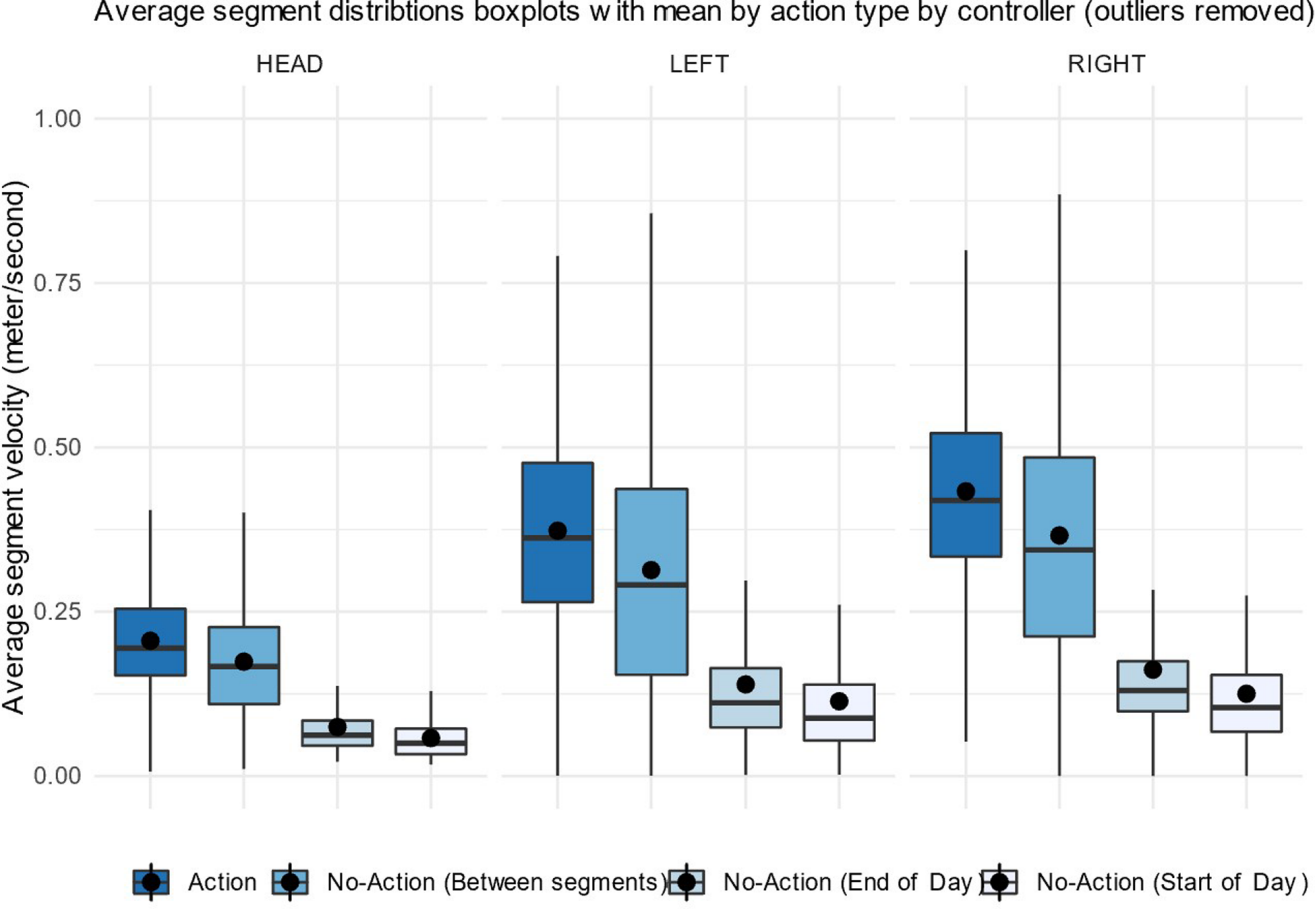
Average velocity (m/s) in movement segments in different accelerometer sensors (head mounted VR device, left-hand and right-hand controllers) by action type.

First, data were evaluated as action vs. no-action segments. As shown in Figure 3, the visual comparison of action data vs. no-action data shows that action data show faster movement on average. This is, of course, a validity check for segmentation only, and result shows that while conducting the activities, participants' average velocity increases. This is natural as participants were first preparing for activities and at the end of day, they stop activities while movement is still recorded. Furthermore, no-action segments between actions are close to action segments in distribution of velocity, as participants were navigating to the next action and were more engaged in system compared to no-action segments at the start and the end of the day.

**Figure 4:**
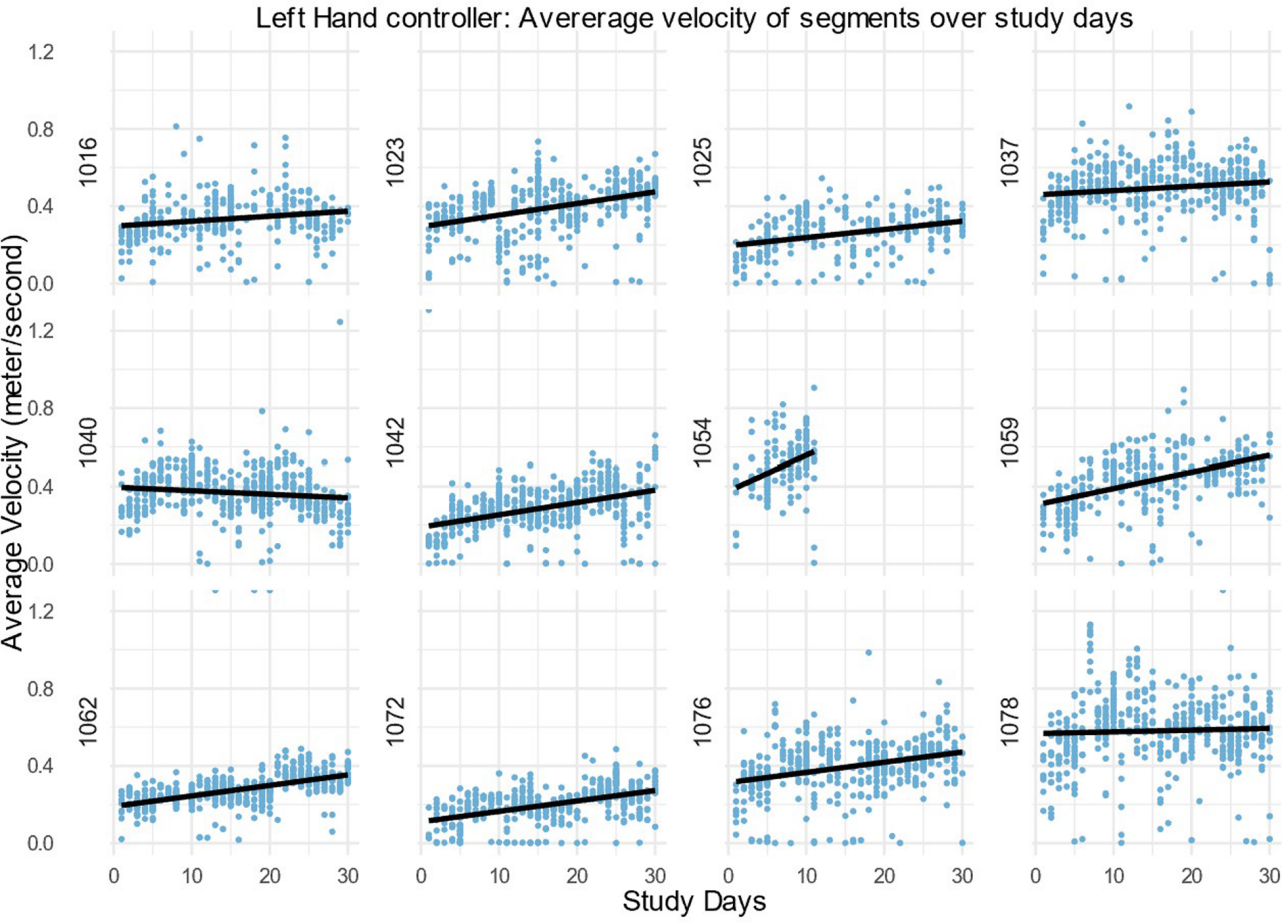
Average velocity in the left-hand controller of all action task segments within the 30 daily sessions (m/s) for all DTxP arm participants, with the regression line over the time.

**Figure 5:**
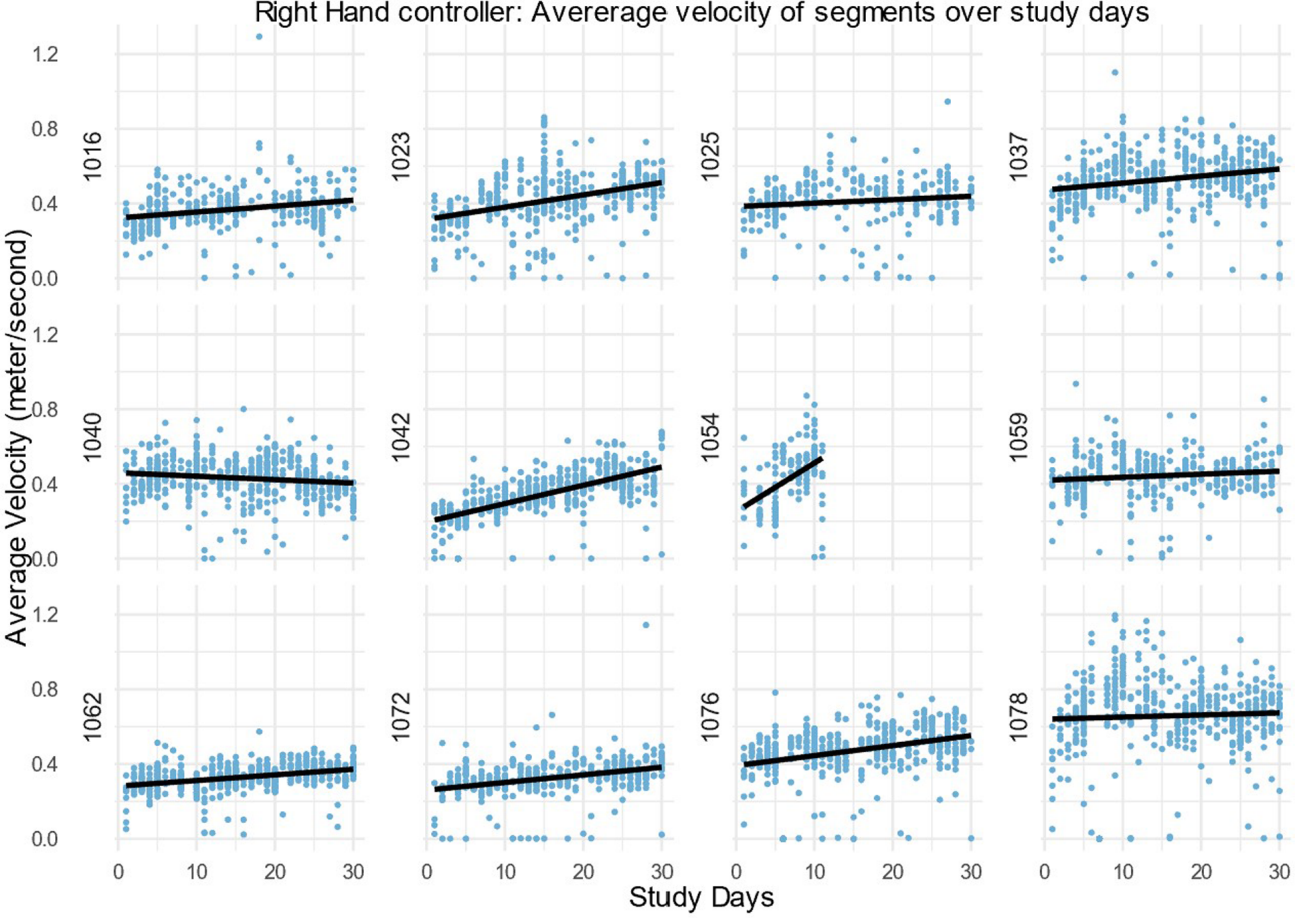
Average velocity in the right-hand controller of all action task segments within the 30 daily sessions (m/s) for all DTxP arm participants, with the regression line over the time.

**Figure 6:**
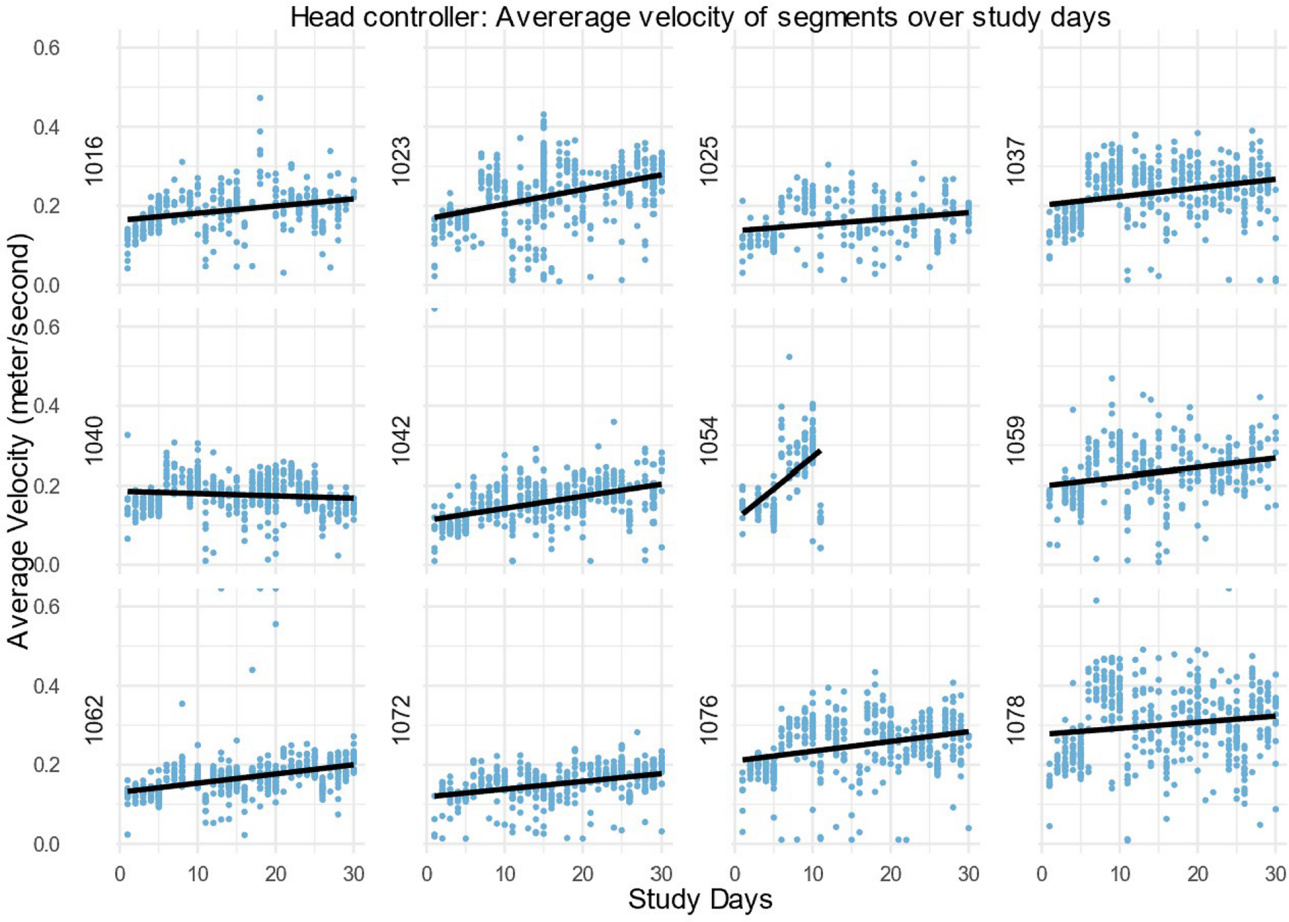
Average velocity in the head controller of all action task segments within the 30 daily sessions (m/s) for all DTxP arm participants, with the regression line over the time.

After this, only the action segments were used to assess the average velocity. Velocity over time was explored by fitting a regression line to the average velocity of action task segments during the study days. The velocity of the left-hand, right-hand and head controllers for every participant is shown in Figures 4–6. For most, the velocity appears to increase over the study. This could be due to many factors, but we suggest that one factor would be participants moving better over time. A similar trend is observable in left hand and head controller movements. There is variance and some obvious outliers in the plots, however, most of the regression lines have an upward tendency.

In this study the interest was participants progression over 30 study days, whereas participant 1054 has only 10 study days. The slope of the regression line for the participant 1054 is three folds more than any other slope. From visual inspection of all patient velocity averages, it is visible that the first study days tend to have more upward progression than rest of the study days. Thus, the missing part of these data could likely cause bias as they are not random by its nature.

Correlations between the slope of average velocity values in the controllers and the change in clinical measurements (TSK, Overall health VAS, EQ5D5L QoL score) during the study were calculated. In Table 1, all the values by participants are shown. Controller values are estimated as the slope of daily change multiplied by study days in VR, i.e., for participant 1,016, the slope of the right controller was 0.0031 and thus the change in velocity from day 1 to day 30 is estimated as 0.095 m/s. Participants had 30 or 29 study days in VR, except participant 1054, who had 11 study days in VR.

**Table 1:** Average velocity change as “slope value * study days” in the controllers (Right, Left, Head) and the change in Clinical assessments (Tampa Scale of Kinesiophobia (TAMPACHG), Overall health Visual analogue scale (VASCHG), EuroQoL-5D-5L QoL score (EQ5DCHG) during the study for each participant in DTxP arm.

SUBJECT	TAMPACHG	VASCHG	EQ5DCHG	Head_chg	Left_chg	Right_chg
1016	−6	20	−4	0.054	0.077	0.095
1023	−6	53	−4	0.111	0.181	0.197
1025	7	−5	4	0.046	0.127	0.055
1037	−12	50	−4	0.066	0.067	0.113
1040	−2	5	0	−0.017	−0.055	−0.055
1042	−8	15	−1	0.088	0.185	0.283
1054	−4	−20	2	0.174	0.200	0.287
1059	−8	15	−3	0.069	0.253	0.049
1062	−11	60	−4	0.070	0.163	0.090
1072	−9	25	0	0.059	0.162	0.122
1076	−8	40	−6	0.074	0.158	0.182
1078	−7	−15	1	0.046	0.027	0.011

The Pearson and Spearman correlations of the controller velocity values, and the clinical endpoints are shown in Table 2, first without participant 1054. Both movement and clinical measurements are strongly correlated within their respective groups. This is expected as the increased activity should be seen as the increased movement velocity in all body parts, and clinical measurements are all validated questionnaires. In addition, the correlations between movement measurements and clinical measurements are found to be on a moderate level. For TSK and EQ5D5L QoL score the increasing movement velocity correlates with a negative change and for Overall health VAS change the correlation is positive. This indicates that increased velocity correlates with improving clinical measures. The association is similar between parametric and non-parametric correlations. To study the robustness of the results, correlations with the full data are examined. When the participant 1054 is included in the data, as also shown in Table 2, the correlations between controllers are strong. However, the correlations between controllers and clinical results are weaker compared to cleaned data, although the Spearman correlations of Head device to clinical measurements remain still on moderate level.

**Table 2:** Pearson and Spearman correlations of the average velocity slopes in the movement controllers and the clinical endpoints in DTxP arm during the study.

Pearson Correlations with 1054 removed from data							Spearman Correlations with 1054 removed from data						
	TampaChg	VASChg	EQ5DChg	HeadChg	LeftChg	RightChg		TampaChg	VASChg	EQ5DChg	HeadChg	LeftChg	RightChg
TampaChg	*						TampaChg	*					
VASChg	−0.59	*					VASChg	−0.59	*				
EQ5DChg	0.71	−0.78	*				EQ5DChg	0.43	−0.81	*			
HeadChg	−0.40	0.55	−0.47	*			HeadChg	−0.45	0.67	−0.66	*		
LeftChg	−0.23	0.37	−0.29	0.79	*		LeftChg	−0.29	0.36	−0.25	0.76	*	
RightChg	−0.34	0.47	−0.40	0.83	0.61	*	RightChg	−0.33	0.56	−0.50	0.79	0.52	*
	
Computed correlation used pearson-method with listwise-deletion.							Computed correlation used spearman-method with listwise-deletion.						
Pearson Correlations with full data							Spearman Correlations with full data						
	TampaChg	VASChg	EQ5DChg	HeadChg	LeftChg	RightChg		TampaChg	VASChg	EQ5DChg	HeadChg	LeftChg	RightChg
TampaChg	*						TampaChg	*					
VASChg	−0.58	*					VASChg	−0.65	*				
EQ5DChg	0.70	−0.82	*				EQ5DChg	0.55	−0.84	*			
HeadChg	−0.17	−0.02	−0.02	*			HeadChg	−0.26	0.28	−0.34	*		
LeftChg	−0.18	0.19	−0.16	0.71	*		LeftChg	−0.15	0.08	−0.04	0.78	*	
RightChg	−0.22	0.11	−0.13	0.86	0.64	*	RightChg	−0.16	0.20	−0.20	0.84	0.57	*
	
Computed correlation used pearson-method with listwise-deletion.							Computed correlation used spearman-method with listwise-deletion.						

### 3.2. Electrodermal activity (EDA) data

Peaks/minute -measurement was compared between DTxP and Sham arms by study day. Overall there are more peaks/minute in early study and then some higher distributions in DTxP arm closer to the end of study. However, the association is not clear.

**Figure 7:**
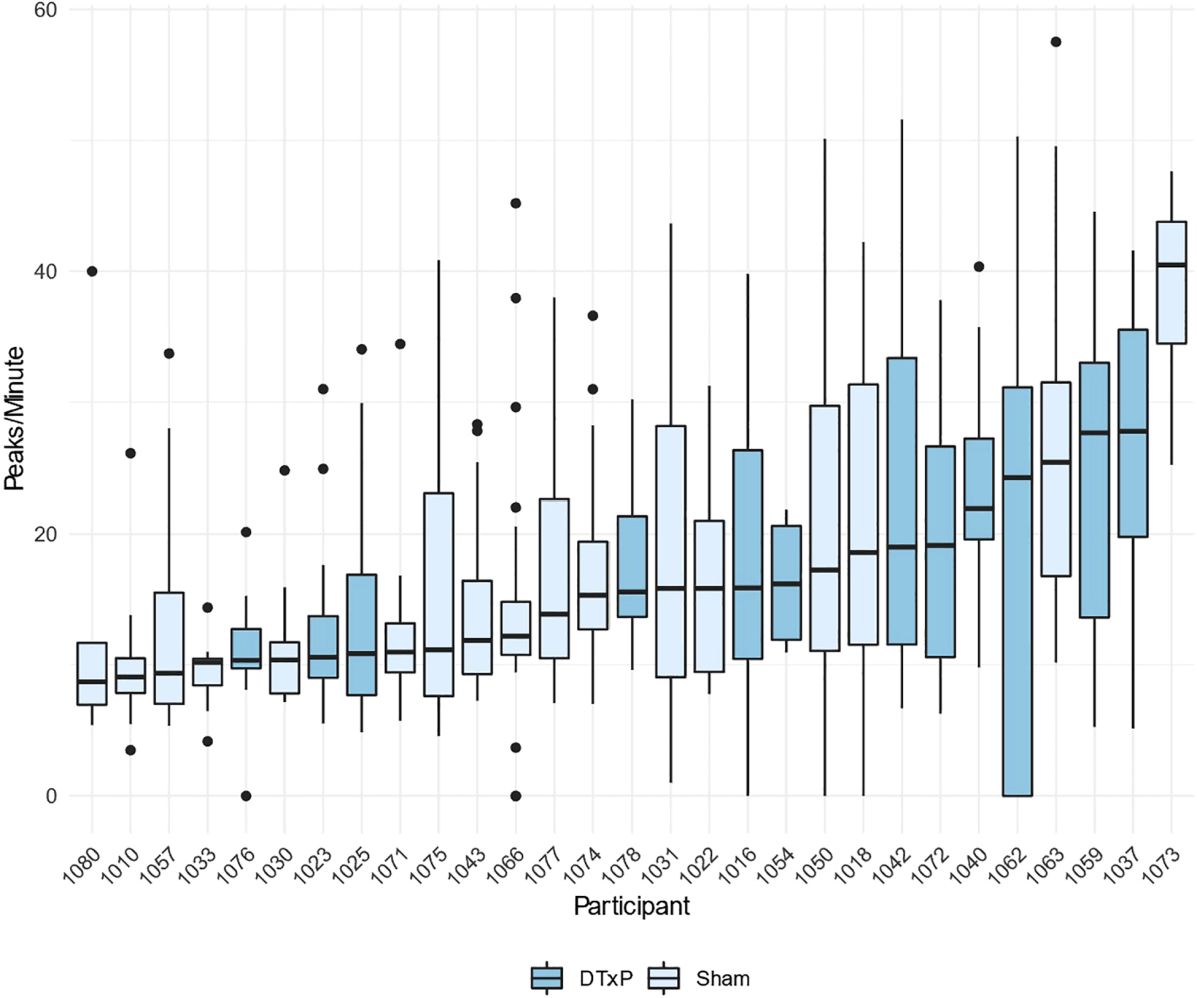
Number of electrodermal activity (EDA) peaks per minute by participant, organized in the ascending order by the median.

When comparing peaks/minute -measurement distribution by participant, we ordered distributions by median (Figure 7). We can see there is some tendency for DTxP arm to have a higher median with larger variability. Sham arm has more participants with clearly narrow distribution with a low median.

### 3.3. Activity data

**Figure 8:**
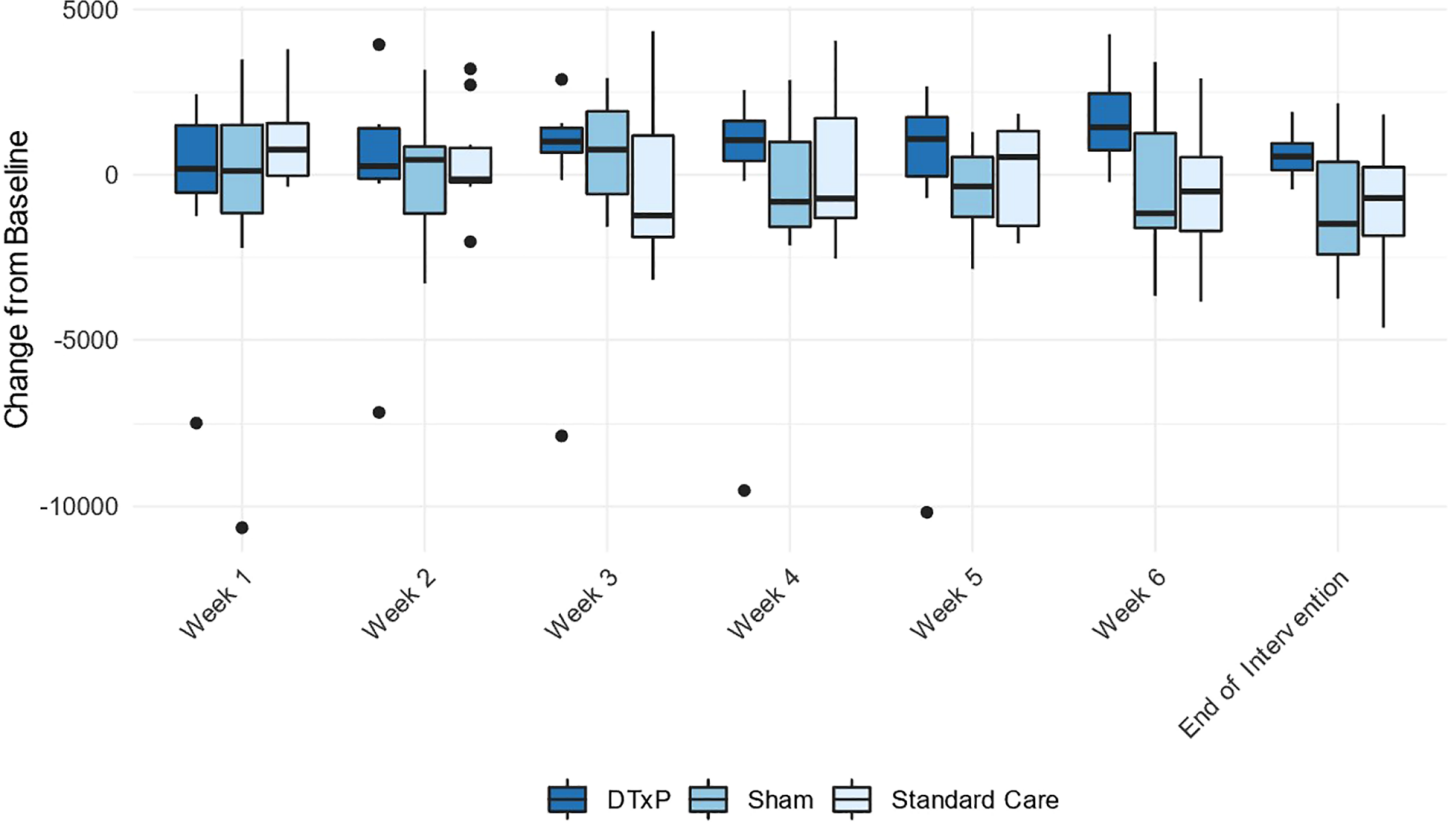
Average steps per day by study week.

Heart rate, steps, and sleep duration aggregates were collected, and steps were visualized. Change in daily mean was calculated and aggregated to weekly measure. Then change distribution over all patients was plotted and analyzed. Some difference in the change in daily steps was observed between groups (Figure 8).

## 4. Discussion/conclusion

Using the opportunity of a clinical trial of a digital therapeutic for chronic low back pain, we collected sensor data from 39 trial participants using Oculus Quest, Empatica Embrace2 and Garmin Vivosmart4 devices. With movement data we analyzed the DTxP arm alone. With electrodermal data we compared data between the DTxP and Sham placebo arms. With activity data, we compared the data between all study arms. We were interested first in how to render and curate large quantities of data into qualitatively valuable clinical information. Further, we were interested in whether any specific data signals could be associated with clinical changes in outcome due to the intervention. When trying to find potential digital biomarkers, exploring the association between variables by using Pearson and Spearman correlations was assessed to be a valid and interpretable method. As the sample size was limited, we decided to use both correlations. Pearson is sensitive to outliers, whereas Spearman has less statistical power as it is using ranks.

Movement data were collected from 12 adults with CLBP, disability, and fear of pain and reinjury undertaking a minimum of 30 sessions of rehabilitation within VR. It did prove possible to manage large datasets from multiple sources collated across participants and across time. Not all data sources were useful. Electrodermal data were noisy and unstable between the sessions, thus unsuitable for further analyses with limited study population. Heart rate data were aggregated over a daytime and so lacking in precision for any meaningful clinical analysis. However fine movement data of head and both hands were more robust for further analysis.

We selected velocity of individual movements over time as a target variable collected from Oculus devices when using the VR, in part because it was possible to segment the data over time and for its relevance to clinical outcomes. We found that for most of the participants the average velocity of all the sensors increased during these segments over the study. This suggests that participant movement improved over time. Furthermore, this longitudinal change correlated with the improvement in clinical endpoint measurements. The increased velocity was found to correlate with decreased TSK and EQ5D-5L QoL score values and increased Overall health VAS values. All data are in line with the improved physical state of the participants. The largest correlation with clinical endpoints was observed with head and right-hand sensors. The latter makes sense since participants were mainly right-handed, and the increase in the movement is likely explained by them using intuitively the dominant hand in activities. What was surprising is the even stronger correlation with the head sensor movement data and clinical endpoints. Yet we are unwilling to say that any body part sensor would not be a potential source for a digital movement biomarker in CLBP. More research is needed but we assume that CLBP patients with kinesiophobia maintain bracing or minimizing of trunk movements in avoidance of feared pain, essentially doing any rapid movements with their back and head.

Positive correlations of velocity with self-report clinical outcomes are the first step in establishing a movement based digital biomarker but there are at least three steps needed to improve confidence in the measure. First, replication is necessary, followed by extension to different interventions and different clinical groups. Second, interpretation of the velocity in this context deserves further consideration. As the segmentation of the movement data was not based on specifically known movements, it is unclear in which movements the change can be seen better than in the others. Thus, collecting data by using more precise metadata about tasks is needed. Third, the characteristics of movement data contains other options than just the velocity. The velocity was chosen due to its simplicity, and because the increase of movement is a desired objective in this target population. Machine learning methods might also enable multidimensional descriptors without a specific value when analyzing movement data.

The study has limitations. For movement data, we used linear regression slope as a measure of change over time. The slope was selected to aggregate the change to a single measure; however, it is not an optimal measure to describe the intermediate changes over time as there could be different phases during follow-up. Further research and more informative data are needed to better quantify the different aspects of movement over time. As mentioned above, because of the nature of the EDA data, any interpretation will remain uncertain. Based on earlier studies, it is however a promising method, but because it is relative to skin temperature and humidity, more data from wider population are needed (7, 27). The activity data analysis was not successful since it contained only aggregated daily data instead of raw data, especially so with heart rate—heart rate variability analysis wasn't possible.

Changes in bodily movement over time appears to offer a clinically meaningful digital biomarker to be exploited in intervention studies aimed at the rehabilitation of adults with chronic low back pain. Further studies would help improve our confidence in this approach, in particular if based on more diverse participants and a greater range of clinical endpoints.

## Data Availability

The raw data supporting the conclusions of this article will be made available by the authors, without undue reservation.
